# Novel Fiber-Optic Ring Acoustic Emission Sensor

**DOI:** 10.3390/s18010215

**Published:** 2018-01-13

**Authors:** Peng Wei, Xiaole Han, Dong Xia, Taolin Liu, Hao Lang

**Affiliations:** School of Instrumentation Science and Opto-Electronics Engineering, Beihang University, Beijing 100191, China; weipeng@buaa.edu.cn (P.W.); summereast@buaa.edu.cn (D.X.); liutaolin@buaa.edu.cn (T.L.); langhao@buaa.edu.cn (H.L.)

**Keywords:** acoustic emission, fiber-optic ring sensor, sensing skeleton

## Abstract

Acoustic emission technology has been applied to many fields for many years. However, the conventional piezoelectric acoustic emission sensors cannot be used in extreme environments, such as those with heavy electromagnetic interference, high pressure, or strong corrosion. In this paper, a novel fiber-optic ring acoustic emission sensor is proposed. The sensor exhibits high sensitivity, anti-electromagnetic interference, and corrosion resistance. First, the principle of a novel fiber-optic ring sensor is introduced. Different from piezoelectric and other fiber acoustic emission sensors, this novel sensor includes both a sensing skeleton and a sensing fiber. Second, a heterodyne interferometric demodulating method is presented. In addition, a fiber-optic ring sensor acoustic emission system is built based on this method. Finally, fiber-optic ring acoustic emission experiments are performed. The novel fiber-optic ring sensor is glued onto the surface of an aluminum plate. The 150 kHz standard continuous sinusoidal signals and broken lead signals are successfully detected by the novel fiber-optic ring acoustic emission sensor. In addition, comparison to the piezoelectric acoustic emission sensor is performed, which shows the availability and reliability of the novel fiber-optic ring acoustic emission sensor. In the future, this novel fiber-optic ring acoustic emission sensor will provide a new route to acoustic emission detection in harsh environments.

## 1. Introduction

Acoustic emission (AE) technology is a type of non-destructive testing (NDT) method. It has been successfully used in aerospace engineering, machinery manufacture, petrochemical industry, and other fields since the 1950s [1]. The conventional piezoelectric (PZT) sensor is currently the most widespread sensor in acoustic emission detection. The PZT AE sensor has several good characteristics, such as high sensitivity and good stability. However, the PZT AE sensor is an electrical sensor and is inevitably susceptible to electromagnetic interference and strong corrosion, and can only operate in a limited temperature range [2]. Furthermore, the PZT sensor and the amplifier must be sufficiently close to avoid serious signal distortion. In addition, the shielded cable for the PZT AE sensor is heavy and hard. However, a fiber-optic ring sensor is totally different. The ring is made from silicon dioxide, is small in size and light weight, and most importantly, it can overcome the shortages of the PZT AE sensor. There have been several types of fiber optic AE sensors, such as fiber Bragg grating (FBG) AE sensors, fiber-optic Fabry–Pérot cavity AE sensors, and fiber-optic ring (FOR) AE sensors. The FBG acoustic emission sensor has high sensitivity, small size and can be embedded in structures [3,4,5]. However, the sensitivity of the FBG AE sensor is different in different directions, which affects its performance. The fiber-optic Fabry–Pérot cavity AE sensor has a wide measurement range and stable character [6]. However, the Fabry–Pérot cavity sensor is difficult and costly to produce, and has low reproducibility efficiency. Several scientists have focused on the FOR AE sensor [7]. The sensor has high sensitivity based on the interferometric phase demodulation method. However, all of the FOR AE sensors that have been proposed are for use in liquid. None has been designed to be applied on a solid specimen. The FOR AE sensor we designed is different from the above fiber optic AE sensors. Compared with FBG AE sensor, our sensor is designed with cylindrical skeleton to eliminate the direction sensitivity. Compared with the Fabry–Pérot cavity AE sensor, the production process of our sensor is simple and the cost is lower, only 2 dollars for each sensor. Also, our sensor can be applied directly to the solid specimen, without liquid [7].

The FOR AE sensor can be demodulated by the homodyne method and the heterodyne method. The homodyne method is easily achieved but can be influenced by low frequency noise [8,9,10]. The heterodyne method is complex and can resist the outside interference of low frequency noise [11,12].

**Figure 1:**
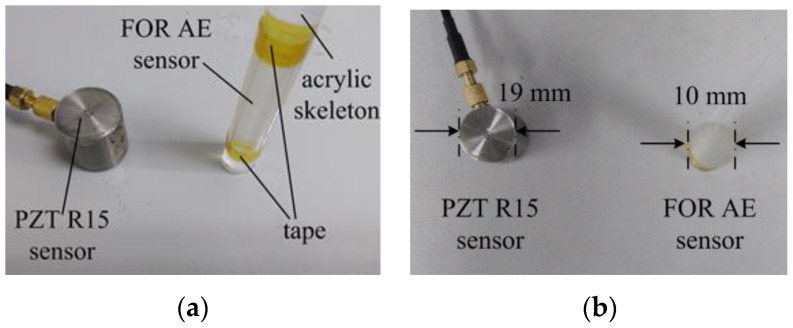
FOR and PZT AE sensor. (**a**) Side view; (**b**) Top view.

In this paper, a novel FOR AE sensor is proposed and used on an aluminum plate. The novel FOR AE sensor is shown in Figure 1.

Figure 1 is the comparison between the FOR and PZT AE sensors. Figure 1a is a side view. Figure 1b is a top view. In Figure 1a, the FOR AE sensor is made of ITU-TG657B3 fiber from YOFC Company, Wuhan, China. The fiber is tightly wound around the skeleton to form a fiber-optic ring. The skeleton is made of an acrylic cylinder with a diameter of 10 mm as shown in Figure 1b. The tape is used to hold the fiber to prevent it from falling off the skeleton.

**Figure 2:**
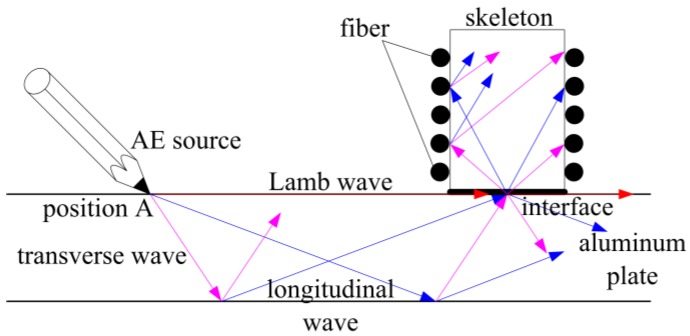
Acoustic emission wave propagation.

In Figure 2 when the AE wave is produced at position A on the aluminum plate by breaking pencil lead, it propagates in the form of Lamb wave, transverse wave, and longitudinal wave along the aluminum plate. Waveform conversion, reflection, and refraction would occur when any form of waves reaching the interface between the AE sensor skeleton and the aluminum plate. Some parts of waves enter the skeleton, and the others remain in the aluminum plate. The waves which enter the skeleton would have waveform conversion, reflection, and refraction again and again. Therefore, the AE waves in the skeleton would be complex waves with multiple forms. However, in this paper, experiment results show that the sensing FOR can be influenced by AE waves. Therefore, the AE waves can be detected by fiber parameter changes.

## 2. Methods

### 2.1. Novel Fiber-Optic Ring Sensor

**Figure 3:**
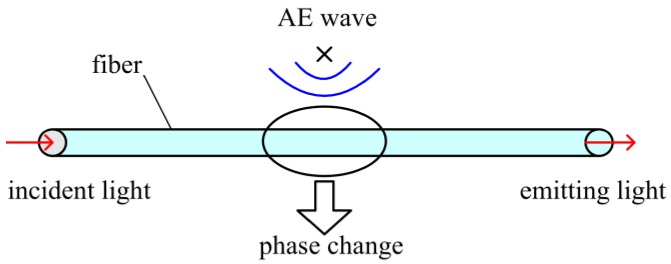
Optical fiber sensing principle.

When light propagates in a fiber, certain characteristic parameters of the light—such as phase—change under the action of an acoustic emission wave [13,14]. Figure 3 shows how the AE wave influences the light phase in the fiber.

**Figure 4:**
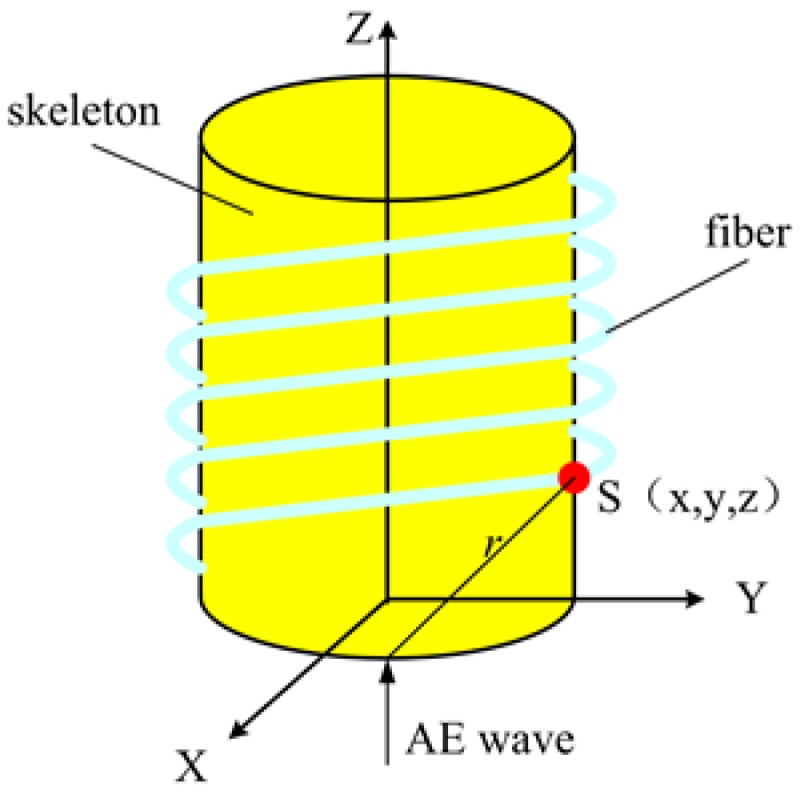
FOR AE sensor.

The FOR AE sensor is a novel sensor where a fiber is tightly wound around a skeleton, as shown in Figure 4. In Figure 4, both the skeleton and the fiber are involved in the AE sensing process.

The phase of light passing through a piece of fiber is shown to be

(1) ϕ=βL

where β is the fiber propagation constant and *L* is the fiber length wound on the skeleton.

The light phase change Δϕ in the FOR AE sensor is due to the acoustic emission wave.

(2) $$\Delta \phi =\beta \Delta L+\Delta \beta L=\Delta \phi_{1}+\Delta \phi_{2}$$

In (2), $\Delta \phi_{1}$ is the phase change due to the axial stretching of the fiber; $\Delta \phi_{2}$ is the change of the propagation constant due to the change of refractive index and fiber diameter.

$\Delta \phi_{1}$ is composed of two parts, shown as

(3) $$\begin{array}{cc} \Delta \phi_{1} & =\beta \cdot \Delta L_{1}+\beta \cdot \Delta L_{2}\\ & =\beta \cdot \varepsilon L+\beta \cdot \Delta L_{2}\\ & =-\frac{\beta L}{E}(1-2\nu )P+\beta \cdot \Delta L_{2} \end{array}$$

where Δ*L*_1_ is the length change of the fiber directly influenced by AE wave, Δ*L*_2_ is the length change of the fiber influenced by skeleton deformation, *ε* is the strain, *E* is the Young’s modulus, *ν* is the Poisson ratio of the fiber, and *P* is the acoustic emission signal.

Based on the theory of the Elasticity [15,16], Δ*L*_2_ can be given as

(4) $$\Delta L_{2}=\int_{0}^{L}s\;dl$$

where *s* is the length change of the infinitely small interval of the skeleton edge. The fiber is tightly wound around the skeleton and the fiber diameter is 250 µm, far less than the diameter of skeleton, which is 10 mm. Thus, it can be considered that *s* is the length change of the infinitely small interval of fiber. We can set up the coordinate system in Figure 4. The origin of the coordinate system is the center of the lower surface of the skeleton, the X and Y directions are in the plane of the lower surface, and the Z direction is perpendicular to the lower surface. Therefore, *s* can be defined as

(5) $$s=\sqrt{{s_{x}}^{2}+{s_{y}}^{2}+{s_{z}}^{2}}$$

where $s_{x}$ is the length change along X, $s_{y}$ is the length change along Y, and $s_{z}$ is the length change along Z.

(6) $$\{\begin{array}{l} s_{x}=\frac{(1+\nu_{m})P}{2E_{m}\;\pi }\sqrt{-\frac{(1-2\nu_{m})x}{r(r+z)}+\frac{xz}{r^{3}}}\\ s_{y}=\frac{(1+\nu_{m})P}{2E_{m}\;\pi }\sqrt{-\frac{(1-2\nu_{m})y}{r(r+z)}+\frac{yz}{r^{3}}}\\ s_{z}=\frac{(1+\nu_{m})P}{2E_{m}\;\pi }\sqrt{\frac{2(1-\nu_{m})}{r}+\frac{z^{2}}{r^{3}}} \end{array}$$

where *x*, *y*, and *z* are the coordinates of the infinitely small interval; *ν_m_* is the Poisson ratio of the skeleton; *E_m_* is the Young’s modulus of the skeleton; and *r* is the distance between the initial position of the acoustic emission wave and the infinitely small interval.

Substituting (5) and (6) into (4), Δ*L*_2_ is obtained by

(7) $$\Delta L_{2}=\frac{(1+\nu_{m})PL}{2E_{m}\;\pi }\cdot K$$

where $K=\sqrt{-\frac{(1-2\nu_{m})x}{r(r+z)}+\frac{xz}{r^{3}}-\frac{(1-2\nu_{m})y}{r(r+z)}+\frac{yz}{r^{3}}+\frac{2(1-\nu_{m})}{r}+\frac{z^{2}}{r^{3}}}$.

In (2), the changing of the fiber diameter has been demonstrated to be negligible [17]; thus, $\Delta \phi_{2}$ can be given.

(8) $$\Delta \phi_{2}=\frac{\beta Ln^{2}}{2E}(1-2\nu )(p_{11}+2p_{12})P$$

where *n* is refractive index of fiber, and *P*_11_ and *P*_12_ are elements of the strain-optic tensor.

Substituting (3), (7), and (8) into (2) generates

(9) $$\Delta \phi =\left\{\frac{\beta L(1-2\nu )}{E}[\frac{n^{2}}{2}(p_{11}+2p_{12})-1]+\frac{\left(1+\nu_{m}\right)}{2E_{m}\;\pi }KL\beta \right\}\cdot P$$

Based on (9), Δϕ is related to *P*, *E_m_*, *L*, and *K*. In other words, Δϕ is related to the AE wave, sensing skeleton, and sensing fiber-optic ring.

The working process of the FOR AE sensor in this paper is different from the FOR AE sensor without skeleton. The skeleton is not simply an acoustic transduction element, it has an important role in AE wave detection. Without it, the FOR AE sensor would not be able to use on the surface of solid.

### 2.2. FOR AE Demodulation Theory

**Figure 5:**
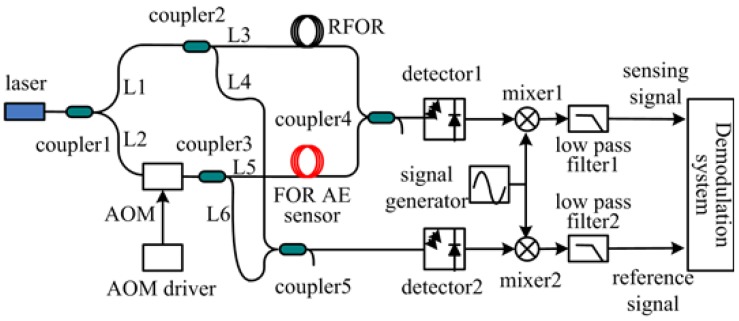
FOR AE system diagram.

To obtain the AE wave, a heterodyne interferometric FOR AE system is built as shown in Figure 5.

In Figure 5, the narrowband light is divided into L1 and L2 by coupler 1, L1 is the fiber reference arm and L2 is the fiber sensing arm. L1 is divided into L3 and L4 by coupler 2. L2 penetrates an acousto-optic modulator (AOM) and is divided into L5 and L6. L3 and L5 respectively penetrate the reference fiber-optic ring (RFOR) and FOR AE sensor; next, an interferometric output is obtained in coupler 4. L4 and L6 produce an interferometric output in coupler 5. The two interferometric outputs respectively penetrate a detector, mixer, and low pass filter. Finally, the reference signal and sensing signal are acquired by the demodulation system. The AOM is driven by an AOM driver with an 80 MHz sinusoidal signal, which shifts the initial light frequency to a higher frequency to avoid the interference of low frequency noise. A 79 MHz sinusoidal signal generated by a signal generator is an input of the mixer. The mixer and low band filter are used to reduce signal frequency so that the signal can be easily acquired by the demodulation system.

In Figure 5, the light field of the FOR AE sensor in L5 is described in (10) under the action of the acoustic emission signal.

(10) $${\vec{E}}_{1}(t)={\vec{A}}_{1}\exp\{j[2\pi (f_{0}+f_{1})t+\delta \phi_{1}(t)]\}$$

where ${\vec{A}}_{1}$ is a light vector, $f_{0}$ is the inherent light frequency, $2\pi f_{1}$ is the 80 MHz shifting frequency from the AOM, and $\delta \phi_{1}(t)$ is the phase change of the light due to acoustic emission signal and low frequency noise.

In Figure 5, the light field of the RFOR in L3 is described in (11)

(11) $${\vec{E}}_{2}(t)={\vec{A}}_{2}\exp\{j[2\pi f_{0}t+\delta \phi_{2}(t)]\}$$

where ${\vec{A}}_{2}$ is a light vector and $\delta \phi_{2}(t)$ is the phase change of the light due to low frequency noise.

An interferometric output in coupler 4 is described as

(12) $$\begin{array}{ccc} I & = & {\left\|{\vec{A}}_{1}\right\|}^{2}+{\left\|{\vec{A}}_{2}\right\|}^{2}+2\|{\vec{A}}_{1}\|\cdot \|{\vec{A}}_{2}\|\cos[2\pi (f_{0}+f_{1}-f_{0})t+\\ & & \left(\delta \phi_{1}(t)-\delta \phi_{2}(t)\right]\\ & = & \left\|{\vec{A}}_{1}{\|}^{2}+\|{\vec{A}}_{2}{\|}^{2}+2\|{\vec{A}}_{1}\|\cdot \|{\vec{A}}_{2}\|\cos[2\pi f_{1}t+\Delta \phi (t)+\phi_{0}(t)\right] \end{array}$$

where Δϕ(t) is the phase change due to the acoustic emission signal, and $\phi_{0}(t)$ is the phase change due to low frequency noise.

The direct current terms in (12) are filtered out by detector 1, the alternating current output is obtained as

(13) $$U_{1}=2\|{\vec{A}}_{1}\|\cdot \|{\vec{A}}_{2}\|\cos[2\pi f_{1}t+\Delta \phi (t)+\phi_{0}(t)]$$

The output of the signal generator is given by

(14) $$U_{sg}=B_{1}\cos(2\pi f_{2}t)$$

where $B_{1}$ is the amplitude and $2\pi f_{2}$ is 79 MHz.

Thus, the output of the mixer is obtained

(15) $$\begin{array}{cc} Y & =U_{1}\cdot U_{sg}\\ & =2\|{\vec{A}}_{1}\|\|{\vec{A}}_{2}\|\cos[2\pi f_{1}t+\Delta \phi (t)+\phi_{0}(t)]B_{1}\cos(2\pi f_{2}t)\\ & =\|{\vec{A}}_{1}\|\|{\vec{A}}_{2}\|B_{1}\{\cos[2\pi f_{1}t+\Delta \phi (t)+\phi_{0}(t)-2\pi f_{2}t]+\\ & \;\cos[2\pi f_{1}t+\Delta \phi (t)+\phi_{0}(t)+2\pi f_{2}t]\}\\ & =B\{\cos[2\pi \Delta ft+\Delta \phi (t)+\phi_{0}(t)]+\\ & \;\cos[2\pi (f_{1}+f_{2})t+\Delta \phi (t)+\phi_{0}(t)]\} \end{array}$$

where $B=\|{\vec{A}}_{1}\|\|{\vec{A}}_{2}\|B_{1}$ and 2πΔf is 1 MHz.

Then, the high frequency portion of Y is filtered out by the low pass filter 1. Therefore, the sensing signal in Figure 5 is obtained

(16) $$Y_{s}=B\cos[2\pi \Delta ft+\Delta \phi (t)+\phi_{0}(t)]$$

The same situation occurs to the reference signal in Figure 5. Therefore, the reference signal is given by

(17) $$Y_{r1}=C\cos[2\pi \Delta ft+\phi_{r}(t)]$$

where *C* is the reference signal amplitude and $\phi_{r}(t)$ is the phase change affected by outside low frequency noise.

**Figure 6:**
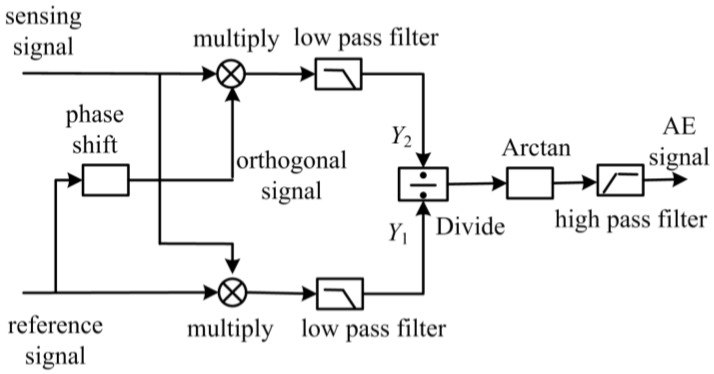
Arctangent demodulation theory.

In Figure 5, the demodulation system is composed of two parts: an acquisition device and demodulation software, which is based on the Arctangent demodulation theory. The demodulation software is shown in Figure 6.

In Figure 6, an orthogonal signal is acquired by shifting π/2 from reference signal.

(18) $$Y_{r2}=C\sin[2\pi \Delta ft+\phi_{r}(t)]$$

Equations (17) and (18) are multiplied by (16), and put through a low pass filter. Next, $Y_{1}$ and $Y_{2}$ are obtained by

(19) $$\begin{array}{cc} Y_{1} & =\frac{1}{2}BC\cos[\Delta \phi (t)+\phi_{0}(t)-\phi_{r}(t)]\\ & =D\cos[\Delta \phi (t)+\phi_{0}(t)-\phi_{r}(t)] \end{array}$$

where $D=\frac{1}{2}BC$.

(20) $$\begin{array}{cc} Y_{2} & =-\frac{1}{2}BC\sin[\Delta \phi (t)+\phi_{0}(t)-\phi_{r}(t)]\\ & =-D\sin[\Delta \phi (t)+\phi_{0}(t)-\phi_{r}(t)] \end{array}$$

Equation (19) is divided by (20).

(21) $$\begin{array}{cc} Y_{3}= & \frac{Y_{2}}{Y_{1}}=-\frac{D\sin[\Delta \phi (t)+\phi_{0}(t)-\phi_{r}(t)]}{D\cos[\Delta \phi (t)+\phi_{0}(t)-\phi_{r}(t)]}\\ & =-\tan[\Delta \phi (t)+\phi_{0}(t)-\phi_{r}(t)] \end{array}$$

By arctangent operation, $Y_{4}$ is obtained

(22) $$Y_{4}=\arctan\;Y_{3}=\Delta \phi (t)+\phi_{0}(t)-\phi_{r}(t)$$

Finally, filtering out the $\phi_{0}(t)$ and $\phi_{r}(t)$ terms by high pass filter, the light phase change Δϕ in the FOR AE sensor can be obtained by

(23) $$Y_{5}=\Delta \phi (t)$$

Substituting (23) into (9), the acoustic emission signal *P* is obtained by

(24) $$Y_{5}=\left\{\frac{\beta L(1-2\nu )}{E}[\frac{n^{2}}{2}(p_{11}+2p_{12})-1]+\frac{\left(1+\nu_{m}\right)}{2E_{m}\;\pi }KL\beta \right\}P$$

## 3. Experiments and Results

### 3.1. FOR AE System Setup

**Figure 7:**
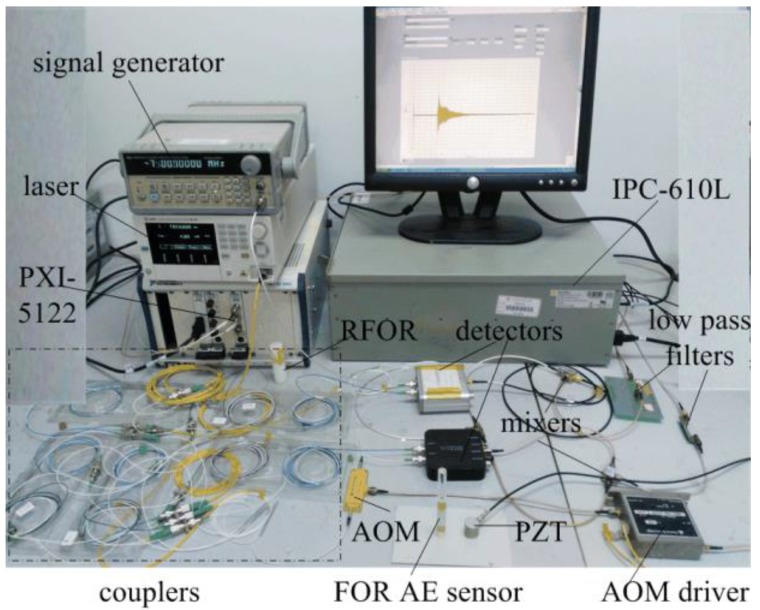
FOR AE system setup.

Based on the above demodulation theory, a FOR AE system is built as shown in Figure 7.

The laser is a TSL-510 by Santec Corporation (Aichi, Japan), the output wavelength of which is 1550 nm, and the optical power of which is adjusted to 0.5 mW. The AOM is a Fibre-Q model, and the AOM driver is a 1080AF-DINA-3.0 HCR model by Gooch & Housego Corporation (Ilminster, UK). The detector is an LPT 200 InGaAs/PIN model detector (Lightpromotech Corporation, Beijing, China). The signal generator is an F80 model (Sample Instrument Technologies Corporation, Nanjing, China). The demodulation system includes an acquisition device and industry computer. The acquisition device is a PXI-5122 model by National Instruments Company (Austin, TX, US). The computer is an IPC-610L by ADVANTECH Company (Taipei, Taiwan). The arctangent demodulation theory is fulfilled by LabVIEW software in the computer.

### 3.2. 150 kHz Sinusoid Experiment

**Figure 8:**
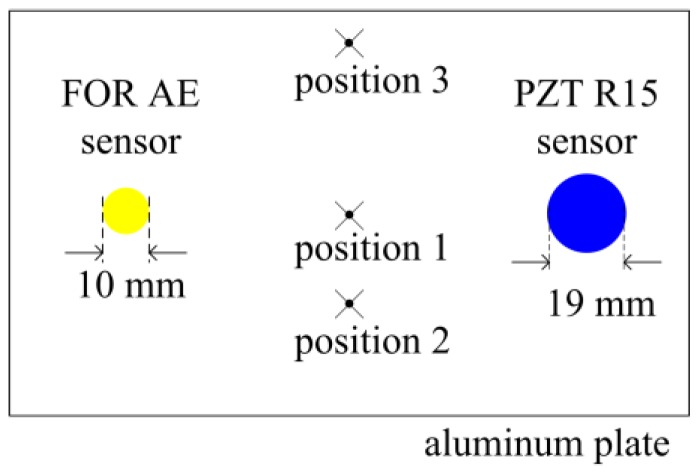
AE sensor experiment.

The FOR AE sensor and PZT AE sensor are glued with Vaseline onto the surface of an aluminum plate, as shown in Figure 8.

In Figure 8, the PZT R15 AE sensor is from Physical Acoustics Corporation (Princeton, NJ, US), whose diameter is 19 mm. The FOR AE sensor is made of acrylic cylinder and fiber, whose diameter is approximately 10 mm.

In Figure 8, a 150 kHz standard continuous sinusoidal signal generated by a FieldCAL from Physical Acoustics Corporation is located in position 1.

**Figure 9:**
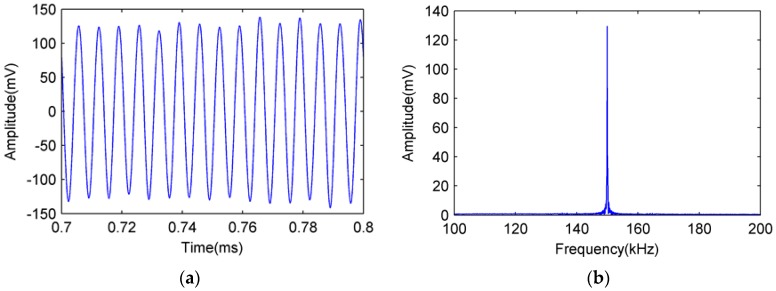
Sinusoidal signal detected by FOR AE sensor. (**a**) Time domain diagram; (**b**) Frequency domain diagram.

**Figure 10:**
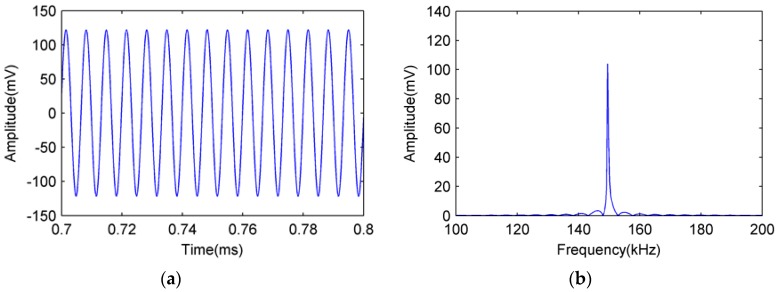
Sinusoidal signal detected by PZT R15 sensor. (**a**) Time domain diagram; (**b**) Frequency domain diagram.

The 150 kHz sinusoidal signals detected by the FOR AE sensor and PZT R15 sensor are shown in Figure 9 and Figure 10, respectively.

Figure 9a is the time domain signal detected by the FOR AE sensor, Figure 9b is the frequency domain signal detected by the FOR AE sensor. Figure 10a is the time domain signal detected by the PZT R15 sensor, and Figure 10b is the frequency domain signal detected by the PZT R15 sensor.

From Figure 9 and Figure 10, we can see that both the FOR AE sensor and PZT R15 sensor can detect a 150 kHz sinusoidal signal well in both the time and frequency domains.

### 3.3. Pencil Lead Breaking Experiment

In Figure 8, when the AE source is a broken lead signal, we can perform the AE detecting experiments by breaking pencil lead. The most representative Hsu–Nielsen broken lead method is used, in which the pencil lead diameter is 0.5 mm, hardness is HB and length is 3.0 mm [18,19].

In Figure 8, there are three positions to break pencil lead. The distance from position 1 to PZT AE sensor and FOR AE sensor is 20 mm; the distance from position 2 to PZT AE sensor and FOR AE sensor is 30 mm; the distance from position 3 to PZT AE sensor and FOR AE sensor is 40 mm. The pencil lead is broken three times at each position and the amplitude of broken lead signals detected by the FOR AE sensor and PZT R15 sensor are shown in Table 1.

**Figure 11:**
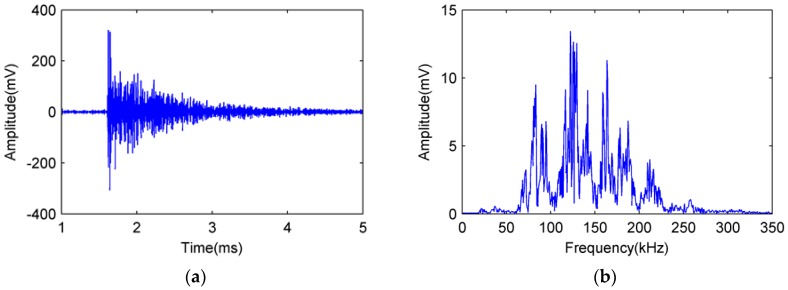
Broken lead signal detected by FOR AE sensor. (**a**) Time domain diagram; (**b**) Frequency domain diagram.

**Figure 12:**
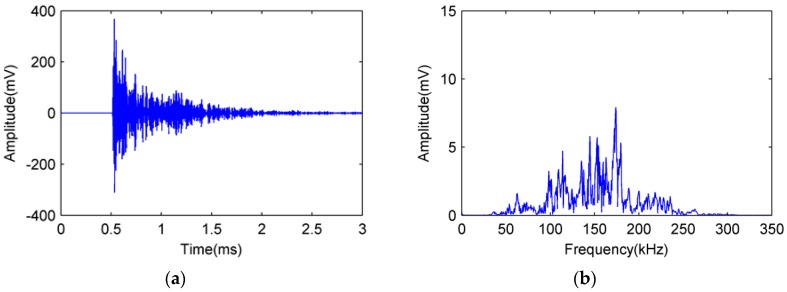
Broken lead signal detected by PZT R15 sensor. (**a**) Time domain diagram; (**b**) Frequency domain diagram.

The broken lead signals detected by FOR and PZT AE sensors in experiment number 4 are shown as Figure 11 and Figure 12, respectively.

In Figure 11 and Figure 12, the broken lead signal detected by the FOR AE sensor is consistent with that detected by the PZT R15 sensor, showing a typical acoustic emission signal [20]. Comparing the amplitude of signal in Figure 11b and Figure 12b, we can see that the signal–noise ratio of FOR AE sensor is better than PZT AE sensor.

From Table 1, we can see that at same position amplitudes of FOR AE sensor are very close. However, at different positions, the amplitude rises as the distance reduces. The same situation happens to PZT AE sensors. Also from Table 1, it can be seen that at the same position the amplitude of FOR AE sensor is very close to the amplitude of PZT AE sensor. That means both PZT and FOR AE sensor can detect the pencil lead signal well.

### 3.4. FOR AE Sensor Direction Sensitivity Experiment

Direction sensitivity is an important characteristic in AE detection. We performed this experiment on an aluminum plate to test direction sensitivity of FOR AE sensor comparing with PZT R15 AE sensor.

**Figure 13:**
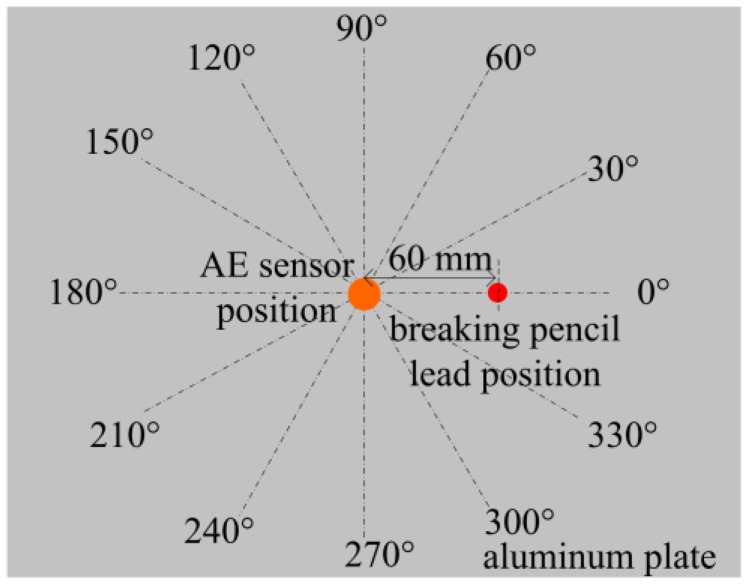
Direction sensitivity experiment.

In Figure 13, AE sensor position is located in the center of the aluminum plate, both the FOR and PZT R15 AE sensor are glued at this position. The distance between AE sensor position and breaking pencil lead position is 60 mm. The breaking lead experiments are done at 12 different angles which range from 0° to 330°. The pencil lead is broken three times at each direction and the average amplitude detected by the FOR and PZT AE sensors are shown in Table 2.

Standard deviation σ can represent the dispersion degree of data, which can be described in (25).

(25) $$\sigma =\sqrt{\frac{1}{N-1}\sum_{i=1}^{N}{\left(x_{i}-\bar{x}\right)}^{2}}$$

where N is 12, $x_{i}$ is the signal amplitude each time, $\bar{x}$ is the average signal amplitude.

Based on Table 2 and (25), standard deviation of FOR AE sensor $\sigma_{FOR}$ is

(26) $$\sigma_{FOR}=0.46dB.$$

Based on Table 2 and (25), standard deviation of PZT AE sensor $\sigma_{PZT}$ is

(27) $$\sigma_{PZT}=0.78dB.$$

Therefore, we can conclude that standard deviations of both FOR and PZT AE sensor are small in each direction, and the direction sensitivities of both AE sensor are consistent in each direction.

### 3.5. FOR AE Sensor Sensitivity Experiment 1

Different FOR AE sensors, skeletons with different materials are made. We select three materials with different Young’s modules: copper, aluminum, and acrylic. All the three materials can propagate the AE signal well, and all the diameters of skeletons are 20 mm, all with sensing fiber lengths of 5 m.

The sensitivity experiments are done as shown in Figure 8. In these experiments, the AE source is a broken lead signal, the distance between the AE source and the FOR AE sensor is 30 mm. The pencil lead is broken three times as the AE source. The average amplitudes of the FOR AE sensor output are shown in Table 3.

From Table 3, we can conclude that the amplitude of the FOR sensor with an acrylic skeleton is larger than those with a copper skeleton and an aluminum skeleton for the same AE source condition. In addition, the amplitude becomes larger as *E_m_* decreases. 

These experiments show that the sensitivity of the FOR AE sensor is related to the Young modulus *E_m_*. However, all the FOR AE sensors with different materials can detect the broken pencil lead signal. That means we can make many different kinds of FOR AE sensors with different materials. For example, in high temperature environments we can choose FOR AE sensor with copper or aluminum skeleton. In this paper we only use three skeletons, but we believe there will be many different kinds of FOR AE sensors with different skeletons in the future. 

### 3.6. FOR AE Sensor Sensitivity Experiment 2

Different FOR AE sensors with different diameter skeletons are produced with a sensing fiber length of 5 m and an acrylic skeleton. There are two types of skeletons with diameters of 20 mm and 10 mm. For the ITU-T G657.B3 fiber (YOFC Company, Wuhan, China) we used, the minimum bend diameter is 10 mm. Smaller than 10 mm would bring heavy bending loss. So we select 10 mm as the minimum skeleton diameter.

The sensitivity experiments are done as shown in Figure 8. In these experiments, the AE source is a broken lead signal, the distance between the AE source and the FOR AE sensor is 30 mm. The pencil lead is broken three times as the AE source. The average amplitudes of the FOR AE sensor signals are shown in Table 4.

In Table 4, the average amplitude detected by the FOR AE sensor become higher as the skeleton diameter reduces for the same AE source.

These experiments show that the sensitivity of the FOR AE sensor is related to the skeleton diameter. However, all the FOR AE sensors with different diameters can detect the broken pencil lead signal. That means we can make many different kinds of FOR AE sensors with different diameters. In this paper, we only use two different diameter skeletons, but we believe there will be many different kinds of FOR AE sensors with different diameter skeletons in the future. 

## 4. Discussion and Conclusions

The FOR AE sensor principle and demodulation method are presented in this paper. The demodulation system is set up to perform experiments and can detect the typical 150 kHz sinusoidal signal and broken lead signal. Comparison with the PZT AE sensor shows the availability and reliability of the novel FOR AE sensor. In addition, the sensitivity experiments demonstrate the performance of the novel FOR AE sensor. Different from other FOR sensors, this novel FOR AE sensor includes two sensing parts: the first is a sensing FOR, and the second is a sensing skeleton. It is qualitatively observed that the Young’s modulus of the skeleton and the diameter of the FOR AE sensor influence the sensitivity of the FOR AE sensor. However, FOR AE sensors work when composed of different materials and at different diameters. That is an outstanding character and could provide infinite possibilities. Based on the theory in this paper, we can make different kinds of FOR AE sensors for different environment. For example, in humid and salt mist environment, the acrylic skeleton FOR AE would be fine, which that is completely different from traditional PZT AE sensors. For a PZT AE sensor, PZT material is the only option.

The combination of coiled fiber and a wide variety of skeleton materials does not only mean a novel method of producing AE sensors, it means novel applications, novel characteristics, and novel cost controls. Without the skeleton, the coiled fiber AE sensor would not threaten the dominant position of traditional PZT AE sensor on a solid surface. A combination of both makes things different. However, this novel FOR AE sensor is just in the beginning. There would be a lot of questions to answer, including optimal design parameters for sensing (skeleton diameter, skeleton height, fiber length, number of coils, etc.) and suitable skeleton materials, coiling, and bonding processes for different applications. However, we do believe that we have opened the door to a colorful AE sensor world. We also believe this unique FOR AE sensor without shielded cable will find applications in many fields, especially in harsh environments and outer space.

## Figures and Tables

**Table 1:** Amplitude of broken lead signal detected by FOR and PZT AE sensors.

Experiment Number	Distance Between Position and Sensor/mm	FOR AE Amplitude/mV	PZT AE Amplitude/mV
1	20	420	425
2	20	417	420
3	20	415	419
4	30	325	338
5	30	310	324
6	30	315	329
7	40	290	300
8	40	295	305
9	40	296	305

**Table 2:** Average amplitude of broken lead signal detected by FOR and PZT AE sensors.

Direction	FOR Amplitude/dB	PZT Amplitude/dB
0°	88.0	89.3
30°	88.2	88.0
60°	87.9	89.7
90°	89.0	90.3
120°	88.8	88.7
150°	89.1	89.7
180°	88.3	88.3
210°	88.1	88.0
240°	88.2	88.7
270°	87.8	88.0
300°	87.8	88.3
330°	87.9	89.3

**Table 3:** Average amplitude of broken lead signal detected by FOR AE sensor.

Skeleton	*E*_m_/G Pa	Average Amplitude/mV
Copper	89	70.0
Aluminum	68	84.5
Acrylic	2.35	156.3

**Table 4:** Average amplitude of broken lead signal detected by FOR AE sensor.

Skeleton Diameter/mm	Average Amplitude/mV
20	156.3
10	316.7

## Abbreviations

The following abbreviations are used in this manuscript.

AE	acoustic emission
NDT	non-destructive testing
PZT	piezoelectric
FBG	fiber Bragg grating
FOR	fiber-optic ring
AOM	acousto-optic modulator
RFOR	reference fiber-optic ring

## References

[1] Shen G. (2015). Acoustic Emission Technology and Application.

[2] Garcia-Souto J.A., Lamela-River H. (2006). High resolution (<1 nm) interferometric fiber-optic sensor of vibrations in high-power transformers. Opti. Express.

[3] Perez I.M., Cui H., Udd E. (2001). Acoustic emission detection using fiber Bragg gratings. Proc. SPIE Smart Struct. Mater..

[4] Mabry N., Banks C., Toutanji H., Seif M. (2011). Acoustic emission felicity ratio measurements in carbon composites laminates using fiber Bragg grating sensors. Proc. SPIE Smart Sens. Phenom. Technol. Netw. Syst..

[5] Wu Q., Okabe Y. (2012). Novel acoustic emission sensor system based on two cascaded phase-shifted fiber Bragg gratings. Proc. SPIE Int. Conf. Fiber Sens..

[6] De Oliveira R., Ramos C.A., Marques A.T. (2008). Health monitoring of composite structures by embedded FBG and interferometric Fabry–Pérot sensors. Comput. Struct..

[7] Garcia-Souto J.A., Posada J.E., Serrano J.R. (2010). All-fiber intrinsic sensor of partial discharge acoustic emission with electronic resonance at 150 kHz. Proc. SPIE Opt. Sens. Detect..

[8] Bua-Nunez I., Posada-Roman J.E., Rubio-Serrano J., Garcia-Souto J.A. (2014). Instrumentation System for Location of Partial Discharges Using Acoustic Detection with Piezoelectric Transducers and Fiber Sensors. IEEE Trans. Instrum. Meas..

[9] Posada-Roman J.E., Garcia-Souto J.A., Serrano J.R., Nunez I.B. (2013). Multichannel ultrasound instrumentation for on-line monitoring of power transformers with internal fiber-optic sensors. Instrum. Meas. Technol..

[10] Posada-Roman J.E., Rubio-Serrano J., Garcia-Souto J.A. (2011). All-fiber interferometric sensor of 150 kHz acoustic emission for the detection of partial discharges within power transformers. Proc. SPIE Int. Conf. Fiber Sens..

[11] Bua-Nunez I., Posada-Roman J.E., Rubio-Serrano J., Garcia-Souto J.A. (2013). Multichannel acquisition system and denoising for the detection and location of partial discharges using acoustic emissions. IEEE Trans. Instrum. Meas..

[12] Posada J.E., Garcia-Souto J.A., Rubio-Serrano J. (2013). Multichannel optical-fibre heterodyne interferometer for ultrasound detection of partial discharges in power transformers. Meas. Sci. Technol..

[13] Liao Y., Li M., Zhang M., Kuang W. (2009). Fiber Sensing Techniques and Applications.

[14] Posada-Roman J., Garcia-Souto J.A., Rubio-Serrano J. (2012). Fiber-optic sensor for acoustic detection of partial discharges in oil-paper insulated electrical systems. Sensors.

[15] Wu J. (2001). Elasticity.

[16] Chen G. (2002). Elasticity.

[17] Hocker G.B. (1979). Fiber-optic sensing of pressure and temperature. Appl. Opt..

[18] (2001). Non-destructive testing—Acoustic emission inspection. http://www.iso.org/standard/34090.html.

[19] (2007). Condition monitoring and diagnostics of machines-Acoustic emission. https://www.iso.org/standard/40686.html?browse=tc.

[20] Yang M. (2010). Acoustic Emission Detection.

